# Open research data in human movement analysis: Swiss-initiated-guidelines for broader scientific communities

**DOI:** 10.1016/j.dib.2025.111884

**Published:** 2025-07-11

**Authors:** Michelle C. Haas, Bettina B. Sommer, Felix Moerman, Simon van Rekum, Eveline S. Graf

**Affiliations:** a Institute of Physiotherapy, School of Health Sciences, ZHAW Zurich University of Applied Sciences, Katharina-Sulzer-Platz 9, P.O. Box, 8401, Winterthur, Switzerland; b Services Research Data, President’s Office, ZHAW Zurich University of Applied Sciences, Gertrudstrasse 15, 8401 Winterthur, Switzerland; c Services Research Data, University Library, ZHAW Zurich University of Applied Sciences, Turbinenstrasse 2, 8400 Winterthur, Switzerland

**Keywords:** Recommendations, Metadata, Data sharing, Kinematics, Kinetics, Electromyography, Current practices

## Abstract

Sharing of data and code leverages transparency and reproducibility of data collection and analysis. However, in biomechanics, such sharing practices are still underdeveloped, possibly due to a lack of knowledge or support. Existing community standards as of today do not yet cover the entire data life cycle and are limited to reporting only one component of movement analysis such as kinematics, kinetics, or surface electromyography, rather than combining these elements. To address this gap, we developed comprehensive guidelines for reporting and sharing human movement data collected with various measurement systems. These guidelines were iteratively developed between January and June 2024, based on requirements which were informed by a survey and workshop within the scientific community. Survey and workshop results highlighted common data sharing practices among movement laboratories, but also significant challenges in standardizing formats, managing metadata, and navigating ethical and legal regulations. The resulting guidelines, structured along the data life cycle, provide detailed recommendations for each stage of the life cycle. Key recommendations include ensuring informed consent for data sharing, maintaining comprehensive metadata, using open formats, and selecting appropriate repositories. Unlike existing standards, which are typically specific to one measurement system, our guidelines integrate and extend upon these recommendations, emphasizing data sharing perspectives tailored to the Swiss and European legal frameworks. To facilitate reporting of metadata, templates are provided. By adhering to these guidelines, researchers can foster a more collaborative, transparent, and impactful scientific community in the field of human movement analysis.

## 1 Introduction

Credibility of research is achieved by fostering transparency and reproducibility of results. However, sharing of data and code, which would leverage transparency and reproducibility of data collection and analysis, is still rare in the field of biomechanics [1]. Moreover, the report “The State of Open Data 2023” by digital science [2] showed that a large proportion of respondents of their cross-disciplinary and internationally conducted survey had never received support with planning, managing, or sharing research data. Only 23 % of respondents received support when sharing data openly. Most of this support was provided by colleagues or supervisors. A small proportion was also provided by the institutional library or in-house institutional experts. The results of this report thus underline the need for more support with planning, managing, or sharing research data. Standards or guidelines could help supporting researchers without the need for one-on-one consultations, thus conserving resources and aid in harmonizing (meta)data standards across the field.

Switzerland’s fragmented landscape of data infrastructure and interoperability frameworks in the field of health and life sciences has been acknowledged as an aggravating aspect by both the Public Health sector and researchers [3]. While centralized frameworks such as the National Coordination Platform Clinical Research or the Swiss Personalized Health Network aim at providing top-down coordination and data flow facilitation, in-depth standards on data processing, curation, and documentation need to be determined bottom-up by the respective communities.

There are standards on reporting of kinematic and kinetic data by the international society of biomechanics [4,5] and consensus statements on reporting electromyography data by the international society of electrophysiology and kinesiology [6,7]. Moreover, for the German-speaking region in central Europe, practice recommendations to support standardization and quality assurance, have been published [8]. However, no comprehensive standards that are applicable to multiple measurement systems exist to support data sharing of researchers in the field of human movement analysis. Thus, preventing the community from adhering to the internationally recognized and widely used FAIR (findable, accessible, interoperable, reusable) principles [9].

In order to advance the data transparency and reproducibility of human movement data, this work aims to provide a set of recommendations for reporting and sharing of such data across various measurement systems.

## 2 Description of Methods

**Fig. 1:**
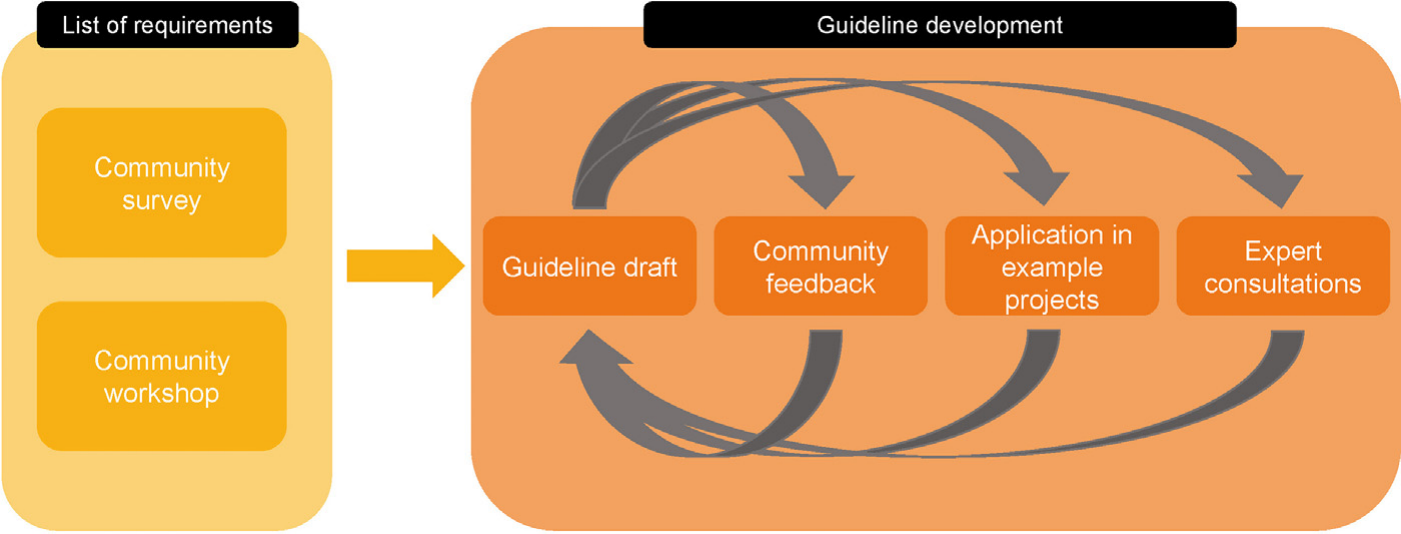
Flowchart of the guideline development process.

Guidelines were developed in an iterative process involving four rounds of feedback and refinement. Iterations were based on a list of requirements which was determined in collaboration with the scientific community (Fig. 1). Iteration rounds incorporated feedback from the scientific community given in writing and verbal discussions, from the local ethics committee, based on new literature as well as by testing the clarity of the guidelines in example projects. The corresponding metadata of these example projects was subsequently published [10].

### 2.1 Definition of requirements

Current open research data practices were identified through an online survey of Swiss movement laboratories in May 2023 and a workshop with staff from four movement laboratories. The survey, consisting of 16 questions, explored data sharing, reuse, formats, software, and documentation. Most questions were multiple-choice. Key interests included data-sharing methods (e.g. public repository, as supplementary material, or personal contact) as well as the motivation and incentives for sharing. Regarding the reuse of data, participants were asked about discipline-specific repositories, search strategies, and experiences with reusing data from others. As standards should be defined by the community, the survey also examined commonly used data formats and software exporting these data formats. Additionally, participants were invited to share their opinion on what components (or information) are essential to comprehensive data documentation. The results of the online survey were summarized descriptively; a statistical analysis was not conducted due to the small number of participants. Respondents expressing interest in further participation (*n* = 6) were invited to join the online workshop.

A three-hour online workshop took place in July 2023 and focused on all aspects of the research data lifecycle, related standard practices, and participants’ experiences with reuse. Four leaders of research groups from different Swiss movement laboratories and with a doctoral degree (affiliated with a university *n* = 1, affiliated with a university hospital *n* = 3) participated in the online workshop. The main objective was to obtain information on personal experiences, but also to discuss the potential of data reuse in the field of human movement analysis. Participants were also asked to report on the format in which the guidelines should be implemented – as a document, interactive website, etc. Based on the information gathered from the survey and the workshop, a list of prioritized requirements was drawn up. Over time, more movement laboratories in Switzerland could be involved in the study, forming a sounding board consisting of senior researchers (*n* = 4) and laboratory leaders (*n* = 8) of 11 movement laboratories. The sounding board was used to obtain feedback in different stages of the project and ensure a wide dissemination and application of the guidelines.

### 2.2 Guideline development

Between January and June 2024, guidelines were developed based on the findings of the survey and workshop. Moreover, consultations from the ethics committee and the institutional data protection officer of the authors helped in clarifying the legal and ethical uncertainties.

### 2.3 Iterative amelioration

Based on feedback from the members of the sounding board, the application of the guidelines in example projects, but also new literature, the guidelines were subsequently and iteratively ameliorated. The corresponding metadata of the example projects generated by following the guidelines is available on Zenodo [10].

## 3 List of Requirements

The anonymously conducted survey achieved a 77 % response rate, with 10 out of 13 Swiss movement laboratories participating. Previous data sharing was reported by 9 out of 10 respondents through public repositories (*n* = 4), publications (*n* = 6), with collaborators (*n* = 3), or personal contacts (*n* = 6). Data was shared with diverse target groups such as with individuals, international researchers, Swiss researchers, other departments within the organization, and organizations both within Switzerland and abroad. Key drivers for data sharing included publishing agreements, personal beliefs towards the benefit of open research data, and funding requirements. In the Swiss community of movement laboratories, the most commonly known repositories are GitHub, Dryad and Zenodo (each *n* = 2). Others like Orthoload, SwissUbase, CAMS-knee, ETH-repository, Olos, and Harvard Dataverse were also mentioned (*n* = 1). To find normative gait data, 60 % of participants would search in publications or directly contact other laboratories, while only 30 % would use public data repositories. There is no standard data format in a movement laboratory, but widely used formats include c3d-files (*n* = 9) for data export and joint angle curves for data presentation. Diversity of used software is high and includes Vicon Nexus (*n* = 10), Matlab (*n* = 8), R (*n* = 5), and proEMG (*n* = 4), among others. The survey also showed that comprehensive metadata is essential for understanding and reusing these data formats.

Four participants from different laboratories attended the online workshop – all acknowledged the growing importance and potential of open research data in recent years. Despite this trend, public availability of data thus far is not or only marginally discussed by the international communities which instead focus on homogenization of data collection methods. The appearance and use of raw data varies by measurement system and data type, making it harder to understand and interpret the data thus increasing the need of a comprehensive documentation. Data archiving procedures also vary by data type. Participants reported that clinical and research data are stored on institutional servers; however, encoding and deletion processes differ between institutions. Clinical data privacy standards can exceed ethical requirements, as experienced by participants, complicating archiving and sharing. Despite many repositories for data publication, complying with legal and ethical regulations remains a concern among participants. There is also a lack of metadata guidelines in movement analysis, which is crucial for data findability. Comprehensive documentation through rich metadata is often highly time-consuming. This could be mitigated by clear guidelines and assistance of data stewards. Each processing step can negatively impact data reuse, making raw data essential but raising more ethical and legal challenges.

The list of requirements (Table 1) was divided into essential definitions or clarifications and optional recommendations.

**Table 1:** List of requirements divided into necessary and optional aspects.

Necessary requirement	Optional requirement
Define…	Recommend…
a standard data format or several formats to ensure readability by humans and machines	a workflow of data publishing after an article has been published
a basic data structure	a workflow of data publishing without additional article publication
a maximal file size to increase feasibility for re-use	a naming convention for files
a standard data format for metadata	which nomenclature should be used e.g. for muscles, bones, tasks, etc.
a set of calculated values that are included by default	how keywords should be selected
which information has to be included as metadata from each phase of the data lifecycle	how different datasets by the same authors or from the same project can be optimally linked
at which level of data processing, the data should be published e.g. as raw data or as graphical illustration	what data is of high value for others
a workflow (descriptive or visual) for internal data reviewing before publication	
Clarify…	
what is ethically required to be allowed to publish data e.g. what has to be included in the informed consent form	
responsibility for data concerning data protection, etc.	
what difference there is regarding the law and ethics when publishing research data from a clinic or a university	
to which level of precision human gait data can be shared	

## 4 Guidelines

**Fig. 2:**
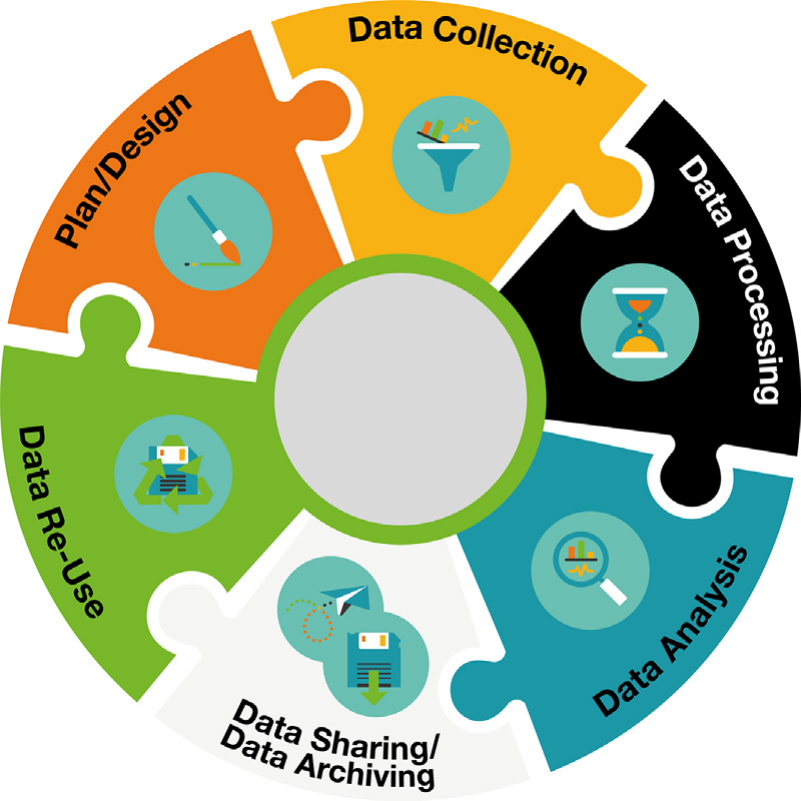
Data life cycle for research data.

The guidelines were developed along the data life cycle (Fig. 2). The detailed Guidelines can be found on Zenodo [11]. Here, the recommendations are summarized for each stage:

### 4.1 Plan / design

This section outlines key considerations when planning a project. If data should be shared later on, ethical and legal aspects need to be addressed, for example, by always including data sharing clauses in the informed consent forms. Comprehensive documentation of the source code should be maintained.

### 4.2 Collect

During data collection, proper documentation must be ensured. Detailed metadata for each participant, including characteristics like age and height, should be collected. General metadata should also be maintained, documenting all software, hardware, data types (e.g. marker-based kinematics, surface EMG (sEMG)), and data formats (e.g. csv-files) used for data collection. Study-specific inclusion and exclusion criteria and task descriptions should be clearly defined and documented. Established guidelines for sensor placement should be followed to ensure data accuracy, and if a marker-based measurement system is used, the marker model should be reported and referenced.

### 4.3 Process

Processing includes the handling of data after the measurement up to the point, where revised data, like angles of a subject, are available. Detailed metadata, including software used and types of data processed, should be reported. Specific processing steps for each data type, including filter parameters used, should be detailed and the analyzed outcomes described. Quality measures, such as manual and automatic checks of data quality, should be included. Version control should be used for source code, along with clear documentation and instructions on how to use the code. Data should be converted to open file formats such as c3d, csv, txt, or json to enhance accessibility and compliance with open data standards.

### 4.4 Analyze

Analysis of data begins once revised data is available. Detailed metadata of data analysis, including software used, types of data analyzed, and information about statistical analysis, should be provided. If the statistical analysis produces code, this code should be provided along with detailed documentation.

### 4.5 Share

While sharing data, several legal, ethical, and quality considerations need to be made. Licenses that align with institutional requirements and the intended use of the data should be selected. A suitable repository should be selected, considering limitations and requirements such as file size, license conditions, and data security, which all vary between different repositories. For example, file size limitations for individual files vary between 2 gigabytes and 5 terabytes while limitations for the total dataset vary between 10 gigabyte and 1 terabyte. Metadata on data sharing, including information on encoding/anonymization methods, processing stages, and data formats, should be documented in an open file format such as csv or txt. Notably, file size limits may impose limitations for projects dealing with big data. Such limitations can be in part addressed by the use of standardized formats for summarized data (reducing the need for sharing raw data). Where the sharing of such summarized data does not suffice, either division of data over multiple files (including metadata on how data was distributed over the files), or the use of specialized data repositories may be necessary.

### 4.6 Archive

Data need to be archived according to legal and ethical aspects. The laws of Switzerland are referenced. Compliance with laws regarding the retention period and encoding of research data should be ensured. Data access should be limited to necessary personnel only.

### 4.7 Reuse

The search for datasets for reuse is addressed in this section. Search methods should combine literature reviews, search engines, and repository searches to find relevant datasets. General repositories like Zenodo should be used for movement data, as there are no FAIR discipline-specific repositories available. The quality of reused data should be assured, and the license should allow for the intended reuse.

## 5 Metadata Templates for R and Python

To facilitate generating metadata documents for movement laboratories, we created a template generator tool, both for the R statistical software and for Python [12]. This tool can be used to generate metadata documents for an individual part of the data lifecycle (data collection – general, data collection – participant information, data processing, data analysis, and data sharing), or for the entire data life cycle. To use the tool, one can download the repository on GitHub or Zenodo, and immediately run the script (depending on preference, R or Python). The tool will then query the user, asking for the relevant information as defined by the MoveD guidelines [11]. The metadata is then stored in a structured csv-file.

## 6 Discussion

This work aimed to provide a set of recommendations for reporting and sharing of data across various measurement systems in the field of human movement analysis. All standards of the International Society of Biomechanics [4,5,[13], [14], [15]] are tailored to specific application areas. They provide recommendations for the definition of various joint coordinate systems such as for the ankle, hip, and spine to be able to report human joint motion in a more uniform way. Moreover, there are recommendations on reporting of intersegmental forces and moments fostering reproducibility of results. These recommendations are predominantly based on marker-based motion capture techniques. However, these specific recommendations are not universally applicable to other measurement approaches such as inertial measurement units, and do not encompass adjacent measurement techniques like sEMG. Conversely, there are well-established standards for reporting sEMG [6,7] which serve as a valuable foundation of the reported guidelines. Combining the knowledge of existing standards for measurement systems commonly used together in clinical practice, such as marker-based motion capture with sEMG, our guidelines integrate diverse aspects and recommendations into a comprehensive overview that is applicable across various measurement systems. They acknowledge and refer to existing recommendations and standards, hence, do not contradict them. Other recommendations such as those from the Clinical Movement Analysis Society of the UK and Ireland [16] provide a solid basis but lack considerations for data sharing. Not all mandatory information listed is essential for understanding the data; also, additional information may be beneficial for comprehending published data. Therefore, our guidelines not only reference existing standards but also enhance them with a focus on data sharing and its specific requirements. No previous guideline included the perspective of data sharing with specific recommendations such as information on licenses, what to consider when choosing a repository to publish data, or legal aspects such as informed consent to publish data. As guidelines were developed for Switzerland, the Swiss legal framework was incorporated. However, regulations for Switzerland and the European Union (EU) are similar, as the Swiss Federal Act on Data Protection was reformed in 2020, to largely align with the GDPR regulations that were adopted for the European Union in 2018 [17,18]. Although the two regulations differ in some respects related to definitions and scopes, the approach to sanctions, and processes for handling violations, regulations are overall highly compatible with each other (see also [19,20] for a full comparison of the two regulatory frameworks by legal experts). For example, informed consent regulations for further use are identical, however a clinical trial master file must be archived for 25 years in the EU, whereas only for 20 years in Switzerland. Other than that, in the referenced legal texts no significant difference exists between the EU and Switzerland. Most sections are generally applicable and do not rely on the legal framework present in a country. Nonetheless, depending on the context/scope of the research (e.g., where date is collected, used, reused), the data may be subject to EU GDPR regulations, Swiss federal regulations (FADP), and/or to Swiss cantonal data protection regulations, which may exist on top of the national data protection regulations (see for example [21,22]). Moreover, the reported guidelines are not only based on the experiences of one movement laboratory but are underpinned by feedback and support from a vast majority of movement laboratories in Switzerland.

The scope of the guidelines is limited by its alignment with Swiss legal regulations and is thus primarily applicable to Switzerland. A further limitation is that the field of open research data is constantly evolving; thus, the guidelines need continual adaptations as new developments might arise or if regulations are updated. It remains challenging to determine which data can be shared when working with human beings. As each research project involves a unique subject group, such as a rare disease or a wider population group, which data can be anonymized and thus shared has to be assessed for each project separately. Hence, no uniform recommendations can be provided.

## 7 Conclusion

In future, guidelines may have a substantial impact on human movement analysis. By facilitating data sharing and reuse, especially using templates, these guidelines can significantly increase the amount of shared data available to the research community. This not only enhances collaboration but also sets a standard for the movement analysis community, promoting consistency and quality in research. Moreover, the potential for data reuse is immense. Human movement analysis is both cost and time-intensive, making it challenging to gather large samples. With well-documented data, researchers can exploit the existing data stock, allowing for larger sample sizes for modelling scenarios. To reach this, open data principles need to be integrated when planning a project. This involves ensuring that informed consent forms include clauses about data sharing, and that comprehensive documentation of metadata and source code is maintained. These steps are crucial for fostering transparency and reproducibility in research.

## Ethics Statement

The authors have read and follow the ethical requirements for publication in Data in Brief and confirm that the current work does not involve animal experiments, or any data collected from social media platforms. The legal requirements of the country where the data were collected do not require an approval by the ethics committee for surveys and workshops when no sensitive or personal data is collected. All data were collected using pseudonymization, data were securely stored and transferred, and informed written consent for survey participation was acquired.

## Declaration of Generative AI and AI-assisted Technologies in the Writing Process

During the preparation of this work the authors used Copilot in order to summarize and increase readability of text passages. After using this tool, the authors reviewed and edited the content as needed and take full responsibility for the content of the published article.

## CRediT authorship contribution statement

**Michelle C. Haas:** Conceptualization, Data curation, Formal analysis, Investigation, Project administration, Validation, Visualization, Writing – original draft. **Bettina B. Sommer:** Data curation, Validation, Visualization, Writing – original draft. **Felix Moerman:** Conceptualization, Data curation, Investigation, Software, Validation, Writing – review & editing. **Simon van Rekum:** Conceptualization, Investigation, Validation, Writing – review & editing. **Eveline S. Graf:** Funding acquisition, Conceptualization, Investigation, Methodology, Validation, Writing – review & editing.
